# Somatostatin Neurons in the Mouse Pontine Nucleus Activate GABA_A_ Receptor Mediated Synaptic Currents in Locus Coeruleus Neurons

**DOI:** 10.3389/fnsyn.2021.754786

**Published:** 2021-10-04

**Authors:** Selena Garcia DuBar, Daniela Cosio, Holly Korthas, Jason P. Van Batavia, Stephen A. Zderic, Niaz Sahibzada, Rita J. Valentino, Stefano Vicini

**Affiliations:** ^1^Department of Pharmacology and Physiology, Georgetown University Medical Center, Washington, DC, United States; ^2^Interdisciplinary Program in Neuroscience, Georgetown University Medical Center, Washington, DC, United States; ^3^Division of Urology, The Children’s Hospital of Philadelphia, Philadelphia, PA, United States; ^4^Department of Anesthesiology and Critical Care, The Children’s Hospital of Philadelphia, Philadelphia, PA, United States

**Keywords:** optogenetic, channelrhodopsin (ChR2), somatostatin (SST), patch-clamp, corticotropin releasing factor (CRF), GABA_A_ receptor, pontine micturition center, arousal

## Abstract

The pontine nuclei comprising the locus coeruleus (LC) and Barrington’s nucleus (BRN) amongst others form the neural circuitry(s) that coordinates arousal and voiding behaviors. However, little is known about the synaptic connectivity of neurons within or across these nuclei. These include corticotropin-releasing factor (CRF^+^) expressing neurons in the BRN that control bladder contraction and somatostatin expressing (SST^+^) neurons whose role in this region has not been discerned. To determine the synaptic connectivity of these neurons, we employed optogenetic stimulation with recordings from BRN and LC neurons in brain stem slices of channelrhodopsin-2 expressing SST or CRF neurons. Optogenetic stimulation of CRF^+^ BRN neurons of *Crf^*Cre*^;chr2-yfp* mice had little effect on either CRF^+^ BRN neurons, CRF^–^ BRN neurons, or LC neurons. In contrast, in *Sst^*Cre*^;chr2-yfp* mice light-activated inhibitory postsynaptic currents (IPSCs) were reliably observed in a majority of LC but not BRN neurons. The GABA_A_ receptor antagonist, bicuculline, completely abolished the light-induced IPSCs. To ascertain if these neurons were part of the neural circuitry that controls the bladder, the *trans-*synaptic tracer, pseudorabies virus (PRV) was injected into the bladder wall of *Crf^*Cre*^;tdTomato* or *Sst^*Cre*^;tdTomato* mice. At 68–72 h post-viral infection, PRV labeled neurons were present only in the BRN, being preponderant in CRF^+^ neurons with few SST^+^ BRN neurons labeled from the bladder. At 76 and 96 h post-virus injection, increased labeling was observed in both BRN and LC neurons. Our results suggest SST^+^ neurons rather than CRF^+^ neurons in BRN can regulate the activity of LC neurons.

## Introduction

Neurons in Barrington’s nucleus (BRN), a pontine micturition center, project to the lumbosacral spinal preganglionic neurons that innervate the bladder. Some BRN neurons are also retrogradely labeled from the locus coeruleus (LC), a major noradrenergic nucleus, suggesting they can impact this system (Valentino et al., 1996). Together, these nuclei amongst others comprise the neural circuitry that is poised to coordinate arousal and voiding behaviors to facilitate micturition.

The diversity in neuronal types, their functional phenotype, and the local synaptic action in the BRN and LC nuclei have not been clearly defined. Past and more recent work has revealed an important GABAergic control of LC (Aston-Jones et al., 2004; Jin et al., 2016; Breton-Provencher and Sur, 2019; Kuo et al., 2020; Breton-Provencher et al., 2021). LC-GABA neurons are also located in the peri-LC and BRN area (Aston-Jones et al., 2004), and optogenetically activating them elicits inhibitory currents in LC neurons that are GABA_A_ receptor dependent (Jin et al., 2016; Breton-Provencher and Sur, 2019; Kuo et al., 2020).

We specifically focused on somatostatin expressing (SST^+^) GABAergic neurons due to their presence in this pontine region and their pivotal role in shaping network dynamics across many brain regions (Ma et al., 2006; Taniguchi et al., 2011; Royer et al., 2012; Lewin et al., 2016; Breton-Provencher and Sur, 2019). We also compare the contribution of CRF neurons to the BRN—LC neural circuitry.

In the BRN, neurons expressing corticotropin-releasing factor (CRF^+^) are of particular importance due to their regulation of bladder function (Hou et al., 2016; Keller et al., 2018; Van Batavia et al., 2021). These CRF^+^ neurons in the BRN are glutamatergic, while those that are CRF^–^ are thought to be either GABAergic or glutamatergic (Hou et al., 2016) and may include SST^+^ GABAergic neurons. This is corroborated by immunohistochemical and *in situ* hybridization studies, which report the co-expression of CRF markers with those of glutamate and GABA neurons in the nucleus (Dabrowska et al., 2013; Hou et al., 2016). In other brain regions, CRF not only co-localizes with glutamate [e.g., in the hypothalamic paraventricular nucleus (Hrabovszky et al., 2005; Füzesi et al., 2016)] but also with other neurotransmitter markers such as GABA and dynorphin (Reyes et al., 2008).

To determine the synaptic connectivity of both SST^+^ and CRF^+^ neurons in BRN and LC pontine nuclei, we combined optogenetic stimulation with whole-cell recordings in brainstem slices of *Sst*^*Cre*^ and *Crf*^*Cre*^ mice (Taniguchi et al., 2011). We tested the hypotheses: (1) SST^+^ neurons are GABAergic and display functional inhibitory synaptic connections with LC or BRN neurons, and (2) CRF^+^ neurons in the BRN influence the activity of other BRN neurons or LC neurons via mono- or poly-synaptic input. To determine whether the pontine neurons we recorded from were part of the neural circuitry that controls the bladder, we retrogradely labeled them by injecting the *trans-*synaptic tracer pseudorabies virus-152 labeled with EGFP (PRV-152 EGFP) into the bladder wall of these mice.

Our results show that light stimulation in *Sst^*Cre*^;chr2-yfp* mice had a significant effect on inhibitory synaptic activity that was more pronounced in the LC neurons than those of the BRN. In contrast, optogenetic stimulation in *Crf^*Cre*^;chr2-yfp* mice failed to reveal a substantial effect on synaptic activity in neurons locally or along the BRN-LC axis. Additionally, we observed that GABA was the primary and functional co-transmitter in pontine SST^+^ neurons, which are more abundant in the BRN than the LC. These observations indicate that SST-BRN neurons can inhibit LC activity.

## Materials and Methods

### Animals

Mice were housed and experiments were performed according to the Animal Care and Use Committee guidelines of Georgetown University. Transgenic mice used were housed under standardized conditions (12 h:12 h light-dark cycle) with food and water available *ad libitum*. The strains examined in this study to genetically identify or activate fluorescent neurons were obtained from The Jackson Laboratory (Bar Harbor, ME, United States). Hemizygous “driver” mice, *Crf*^*Cre*^ [JAX # 012704 (B6(Cg)-CRF^TM 1(cre)*Zjh*^/J)] (Taniguchi et al., 2011; Cusulin et al., 2013; Silberman et al., 2013; Hou et al., 2016; Keller et al., 2018), *Sst^*Cre*^* [JAX #013044 (Ssttm2.1(cre)Zjh)] (Taniguchi et al., 2011; Chen et al., 2015), or *Pv*^*Cre*^ [JAX:008069 (B6;129P2-Pvalb^TM 1(cre)Arbr^/J)] (Hippenmeyer et al., 2005) were bred with either *tdTomato* “*rosa26* floxed-stop” Ai14, JAX # 007914 (Madisen et al., 2010), or *chr2-tdTomato*; “Ai27D”; JAX# 012567 homozygote “reporter” mice, or *chr2-yfp* [ChR2H134R-EYFP, Ai32, JAX# 012569 (Madisen et al., 2012)].

### Viral Injections

All surgeries for virus injections were performed under isoflurane anesthesia (4–1.5% MAC) in accordance with the National Institutes of Health (Bethesda, MD, United States) guidelines and with the approval of the Georgetown University Institutional Animal Care and Use Committee.

#### Pseudorabies Virus

Mice (4–8 weeks *Sst*^*Cre*^;*tdTomato* or *Crf^*Cre*^;tdTomato*) were injected with pseudorabies virus (PRV-152 EGFP, a gift from Dr. Lynn Enquist, Princeton University Center for Neuroanatomy with Neurotropic Viruses, NIH grant number P40RR018604) in the wall of the urinary bladder in a manner previously described by us for the stomach (Lewin et al., 2016). Briefly, after a surgical depth of anesthesia was confirmed, an abdominal laparotomy was performed via a midline abdominal incision along the *linea alba*. The bladder was exposed and gently retracted from the surrounding tissue using a customized glass rod. Next, using a 10 μl Hamilton syringe with a 30-gauge stainless steel needle, PRV-152 EGFP (∼5–10 μl) was slowly injected into the detrusor muscle of the dome of the bladder. On completion, the needle was kept in place for at least 1 min after which it was slowly withdrawn. Leakage of the virus was rare, if any, as seen under a dissecting microscope (total magnification of 40×). The bladder was placed back inside the peritoneal cavity and the abdominal incision closed in a two-step procedure using a 5-0 vicryl suture. After postsurgical medication that consisted of Ringer’s solution (0.5–1 ml; SC) and Buprenorphine SR Lab (0.5–1.0 mg/kg; SC; ZooPharm, CO, United States), animals were returned to their home cages where they were allowed to recover for 72–96 h. Mice were anesthetized with Euthasol^®^ (150 mg/kg IP) and perfused intracardially with phosphate-buffered saline [PBS; in mM: NaCl (137), KCl (2.7), Na_2_HPO_4_ (10), KH_2_PO_4_ (1.8); pH7.4] followed by 4% paraformaldehyde in PBS. All brains were post-fixed for 2 h and then cut on a vibratome (Leica 1000) into 100 μm sections, which were then mounted on glass slides for imaging.

#### Adeno-Associated Virus (AAV)

Mice (4–8 weeks *Sst^*Cre*^* or *Crf^*Cre*^*) were injected with rAAV5-EF1a-DIO-EYFP and AAV5-EF1a-DIO-hChR2(H134R)-EYFP virus (0.2–0.5 μl/injection; UNC core AV4310i) that enabled the expression of EGFP or ChR2-EYFP in *Cre* expressing neurons in the pons. Following a limited dorsal craniotomy, microinjections of the virus were made bilaterally via a single-barreled glass pipette (outer tip diameter ∼35–50 μm; FHC, ME, United States) that was connected to a PE-50 tubing. The stereotaxic coordinates for the injections into the pons from bregma were: AP = −5.42 mm; ML = 0.6 mm; and DV = −3.6 mm. All microinjections were made over ∼1–2 min, and on completion were left in place for a further 5 min before being retracted, the wound covered with sterile Gelfoam^®^, and the skin sutured with a 5-0 vicryl suture. Post-surgery, all animals received Ringer’s solution (0.5–1 ml; SC) and Buprenorphine SR Lab (0.5–1.0 mg/kg; SC; ZooPharm, CO, United States) after which they were returned to their homes and allowed to recover for 2–3 weeks before brains were fixed with PFA for imaging (see above) or prepared for electrophysiology.

### Slice Preparation

Male or female mice were sacrificed by decapitation in agreement with the guidelines of the AMVA Panel on Euthanasia and the Georgetown University Animal Care and Use Committee. Acute brain-slice preparations were prepared as described previously (Lewin et al., 2016; Partridge et al., 2016). Following dissection, brains were rapidly placed in ice-cold slicing solution containing (in mM): NaCl (85), KCl (2.5), CaCl_2_ (1), MgCl_2_ (4), NaH_2_PO_4_ (1), NaHCO_3_ (25), glucose (25), and sucrose (75), osmolarity 325 mOsm. Recordings from 250 μm coronal slices of the pons containing the BRN and the LC were performed at room temperature (23°C) in artificial cerebrospinal fluid (ACSF) containing (in mM): NaCl (124), NaHCO_3_ (26), dextrose (10), KCl (4.5), CaCl_2_ (2.0), Na_2_HPO_4_ (1.2), and MgCl_2_ (1), osmolarity 325 mOsm. Epi-fluorescent excitation of the tissue allowed neuron selection using a 60× water immersion objective.

### Pharmacology, Electrophysiology, and Optogenetics

Recording pipettes were filled with either potassium chloride (KCl) or potassium gluconate (Kgluc) solutions. The KCl solution contained (in mM): KCl (145); HEPES (10), EGTA (5), ATP.Mg (5), and GTP.Na (0.2), osmolarity 305 mOsm; adjusted to pH ∼7.20 with KOH. For Kgluc-based internal solutions, KCl was substituted with potassium gluconate (145 mM). Stock solutions of bicuculline methobromide (BMR), tetrodotoxin (TTX), 2,3-dioxo-6-nitro-1,2,3,4-tetrahydrobenzo[f]quinoxaline-7-sulfonamide (NBQX), and 4-Aminopyridine (4-AP) were prepared in water and diluted 1:1000 in ACSF. All drugs were purchased from Abcam Biochemical (Cambridge, MA, United States). The final concentrations of drugs used were: BMR (25 μM), NBQX (5 μM), TTX (1 μM), and 4AP (30 μM).

Recording electrodes (3–4 MΩ) were made from glass capillary tubes (Drummond Scientific Company, Broomall, PA, United States, Cat. #5-000-2050) using a vertical puller (PP-83, Narishige, Tokyo, Japan). All recordings were acquired using a MultiClamp 700B amplifier that was coupled to a Digidata 1440A (Molecular Devices, Sunnyvale, CA, United States). All signals were sampled at 5 kHz and low-pass filtered at 2 kHz for data analysis. Patch-clamp recordings were performed in the cell-attached mode to measure extracellular spikes and whole-cell mode to measure action potentials in current clamp and synaptic current in voltage clamp.

Coronal slices from channelrhodopsin expressing mice were acutely prepared from postnatal day 17 to 2-month-old animals. Recordings from virus-injected animals were made in coronal slices prepared from animals 2–3 weeks after surgery. Epi-fluorescent excitation of the tissue allowed neuron selection using a 60× water immersion objective. Slices were excited with 488 nm light from X-Cite 120 LED (Excelitas Technologies, Waltham, MA, United States) with a maximal light intensity (15–800 μW) adjusted to prevent loss of voltage-clamp (V_*hold*_ = −60 mV). The diameter of the area exposed to optogenetic stimulation under the 60× objective was <100 μm and encompassed the whole BRN-LC. All experiments were performed at room temperature (22–24°C). Access and input resistance were monitored periodically with a 15 ms, −5 mV test pulse applied 30 ms before the onset of light stimulation. Those neurons in which the access resistance changed by >10% were discarded from the final population of cells for statistical analyses. Light stimulations did not alter the passive membrane properties as assessed by the test pulse.

### Immunostaining

Brain sections were prepared from adult male or female mice aged ∼12 weeks. After isoflurane anesthesia, animals were intracardially perfused with PBS, which was followed by buffered 4% PFA. The brains were then removed and stored overnight in the 4% PFA after which they were transferred to a 20% sucrose PBS solution.

Corticotropin releasing factor immunostaining was performed in a manner and with validation previously reported by one of us (Valentino et al., 2001). Briefly, frozen 30 μm coronal sections were cut on a cryostat and collected in wells in PBS. Sections were incubated in 0.75% H_2_O_2_ for 20 min and then rinsed 3 × 10 min with PBS containing 0.3% Triton-X and 0.04% bovine serum albumin. Sections were incubated with rabbit anti-CRF (1:1000, Dr. Wylie Vale, the Salk Institute for Biological Studies, La Jolla, CA, United States). Following three PBS-TX-BSA rinses (10 min each), sections were incubated in donkey anti-rabbit conjugated to FITC (Jackson ImmunoResearch Inc., West Grove, PA, United States) secondary antibodies for 90 min at room temperature.

For tyrosine hydroxylase (TH) staining frozen 100 μm coronal sections from S*st^*Cre*^;tdTomato, Pv^*Cre*^;tdTomato* or *Crf^*Cre*^;tdTomato* mice were permeabilized with PBS containing 0.5% Triton X-100 and 10% normal goat serum (NGS) for 2 h. Sections were then incubated in rabbit polyclonal anti-TH (1:2000, AB152, Sigma-Aldrich) or mouse monoclonal anti-TH (1:200, MA1-24654, Invitrogen) antibodies in PBS containing 0.1% Tween 20, 1% NGS, 1% bovine serum albumin, and 0.05% sodium azide for 24 h at 4°C. Following six washes for 15 min each, sections were incubated with goat anti-rabbit Alexa Fluor 488 (1:1000, A-11034, Invitrogen), goat anti-rabbit Alexa Fluor 647 (1:1000, A32733, Invitrogen), or goat anti-mouse Alexa Fluor 647 (1:1000, A32728, Invitrogen) secondary antibodies. Sections were then washed in PBS, mounted, and cover-slipped with Fluoromount G (SouthernBiotech, Birmingham, AL, United States).

For GAD staining, 100 μm coronal sections were permeabilized with PBS containing 3% BSA and 10% normal goat serum for 1 h at room temperature. Sections were then incubated in mouse monoclonal anti-GAD67 (1:200, MAB5406, Sigma-Aldrich) antibody in PBS containing 3% BSA and 10% normal goat serum for 48 h at room temperature. Following three washes for 30 min each, sections were incubated with goat anti-mouse Alexa Fluor 647 (1:500, A32728, Invitrogen) secondary antibody. Note that for anti-GAD staining specifically, no detergent was used for any step (Dimidschstein et al., 2016; Breton-Provencher and Sur, 2019). Sections were then washed with PBS and mounted for imaging as above. Confocal image z-stacks were acquired and processed into composite 12–20 image z-stacks with ImageJ software.

### Confocal Imaging

Confocal z-stacks were acquired with either a 20× or 40× objective at 1 μm step size at a resolution of 1024 × 1024 pixels with a ThorLabs, Inc., Imaging resonance laser scanning confocal (Sterling, VA, United States) that was equipped with an argon laser (λ = 488 nm and 561 nm) and a HeNe laser (λ = 642 nm), integrated on a Nikon Eclipse FN1 upright microscope. The confocal images obtained were converted to “z-projection” stacks with ImageJ software (Bethesda, MD, United States) for display purposes. Cell counts were made with a region of interest (ROI) selected with Image J from labeled neurons in individual planes in each stack.

### Fluorescence *in situ* Hybridization

Serial brainstem 10 μm thick frozen coronal sections through BRN were cut using a microtome from 8 to 12-week-old wild type mice (C57BL/6J, JAX# 000664). Separate DNA oligonucleotide probes for CRF and SST were coupled to an amplification system utilizing two different fluorophores (RNAscope^®^ from Advanced Cell Diagnostics, Inc., Hayward, CA, United States) to visualize mRNA expression. Percentage of CRF^+^ and SST^+^ neurons were assessed from total nuclear counts defined by staining with 4′,6-diamidino-2-phenylindole (DAPI).

### Statistics

Data were analyzed using GraphPad Prism 9 or Microsoft Excel software. Results are expressed as the mean ± SEM. All values were tested for normality before proceeding to the specified test listed in the text or figure legend. Significance was based on *p* ≤ 0.05 (^∗^), *p* ≤ 0.01 (^∗∗^), *p* ≤ 0.001 (^∗∗∗^), or *p* ≤ 0.0001 (^****^). Figures were prepared with ClampFit, ImageJ, Microsoft PowerPoint, Adobe Photoshop CS, GraphPad Prism 9, or Microsoft Excel software. While both age and sex are biological variables of interest, they were not of primary concern for this manuscript. Thus, we collapsed across age and sex to maximize statistical power for our comparisons of primary interest.

## Results

### Somatostatin Expressing and Corticotropin Releasing Factor Expressing Neurons in the Barrington’s Nucleus and Locus Coeruleus

**FIGURE 1:**
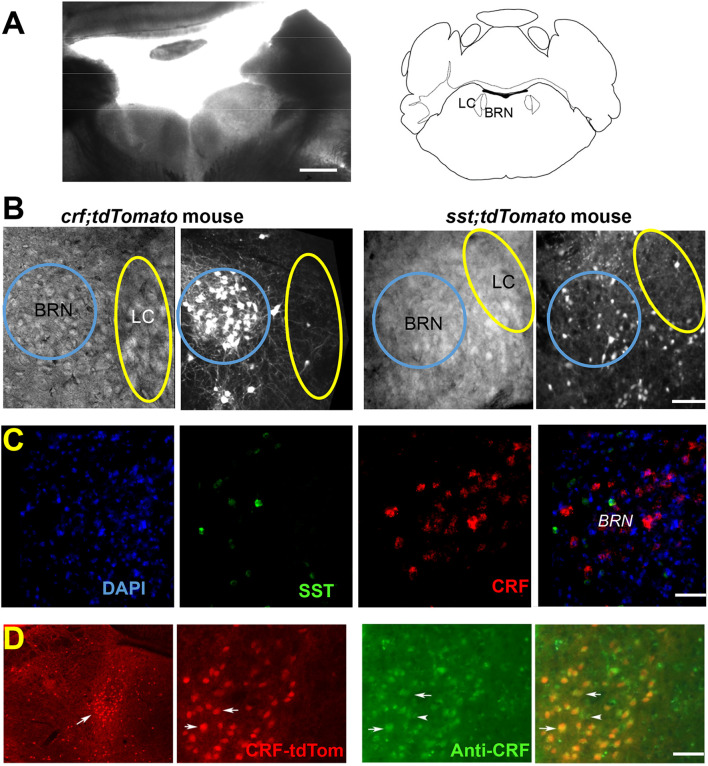
Identification of neurons in Barrington’s Nucleus/Locus Coeruleus area. **(A)** Low magnification bright-field image shows the location of BRN and LC in an example coronal slice from a *Crf^*Cre*^;tdTomato* mouse at postnatal day 28 together with the Brain Atlas map, adapted from Franklin and Paxinos (1997). Scale bar = 0.5 mm. **(B)** DIC and fluorescence tdTomato expression images. *Crf^*Cre*^;tdTomato* expressing neurons (left) are seen as a bright cluster of high neuronal densities in the BRN. tdTomato expression in SST^+^ neurons is seen at a lower density *(right)* in an example slice from *Sst^*Cre*^;tdTomato* mouse. The areas of the BRN and LC are indicated by a blue or yellow circle, respectively. Scale bar = 50 μm. **(C)** RNAscope^®^ *in situ* hybridization of CRF mRNA (red) and SST mRNA (green) does not overlap in BRN. First panel DAPI (blue), second panel CRF (red), third panel SST (green), and fourth panel overlap of all three channels. Scale bar = 50 μm. **(D)** Immunodetection of CRF in BRN neurons (Barr, white arrow) expressing tdTomato. Low magnification images, illustrate the extent of red fluorescent CRF^+^ neurons in sections containing the BRN (Barr, white arrow) and the LC (left). Anatomical distribution is compared at higher magnification between CRF mediated tdTomato expression labeled CRF-tdTom (red), anti-CRF antibody staining (green), and merged images (yellow), indicating accurate transgene expression. Example co-labeled cells indicated by white arrows. Scale bars 1 mm (left) and 50 μm (all other panels).

Mice that express Cre-recombinase in neurons directed by the promoter for the CRF gene have been previously used to study CRF immuno-positive neurons in the BRN (Hou et al., 2016). In addition, Breton-Provencher and Sur (2019) reported the presence of GABAergic neurons in the BRN-LC that innervate the LC using *Gad^*Cre*^* mice. We extended these results (Taniguchi et al., 2011) to establish the potential synaptic connectivity between BRN and LC neurons in *Crf^*Cre*^* mice, and in mice where the expression of Cre-recombinase was directed by the promoter for the *Sst* gene. As illustrated in Figures 1A,B, the distribution of both CRF^+^ and SST^+^ neurons was predominantly seen in BRN/LC area. Whereas *Crf^*Cre*^;tdTomato* expressing neurons were mostly present as dense bright clusters in the BRN (Figure 1B, *left*), *Sst^*Cre*^;tdTomato* expressing neurons in BRN were fewer and sparsely clustered in the nucleus (Figure 1B, *right*). In contrast to BRN, few CRF or SST neurons were observed in the LC. Immunostaining against tyrosine hydroxylase displays the location of LC neurons in respect to the CRF^+^ (Supplementary Figure 1A) or SST^+^ (Supplementary Figure 1B) neurons in the BRN *Sst^*Cre*^;tdTomato* expressing neurons were also found in the area surrounding the LC as it has been reported for the distribution of GABA positive neurons (Breton-Provencher and Sur, 2019). Investigation for the presence of parvalbumin positive (PV^+^) GABA neurons in the BRN-LC area using *Pv^*Cre*^;tdTomato mice* (Hippenmeyer et al., 2005), failed to reveal the presence of these neurons (Supplementary Figure 1C). The edges of the ventricle were, however, lined up with parallel PV^+^ axonal fibers. Upon inspection of slices from the BRN/LC area in a *Sst*^*Cre*^: *tdTomato* mouse, we observed that SST^+^ neurons were co-localized with GAD IHC staining (Supplementary Figure 1.2).

*In situ* hybridization with RNAscope^®^ with fluorescent RNA probes for CRF and SST mRNA in 3 wild type mice confirmed the expression of both probes in BRN (Figure 1C). CRF^–^ expressing neurons were 42% of total DAPI fluorescent cells; SST neurons were 4.7% of neurons. No overlapping was observed (*n* = 1986 neurons; 3 mice; Figure 1C). As regards to CRF expressing neurons, immunostaining with antibodies revealed co-expression of somatic tdTomato with that of CRF immunoreactivity (Figure 1D). Of the BRN neurons examined, (*n* = 153 neurons/3 sections/2 mice), CRF immunoreactivity was observed in ∼99% of the tdTomato neurons. Conversely, 91% of soma in BRN containing CRF immunoreactivity co-expressed with tdTomato.

Cell-attached recordings from LC neurons in brain slices from *Crf*^*Cre*^ or *Sst*^*Cre*^ mice were comparable to those previously reported (Supplementary Figure 2A, *upper left*; Andrade et al., 1983; Williams et al., 1984; Jedema and Grace, 2004; Breton-Provencher and Sur, 2019). In pontine slices from the BRN of *Crf*^*Cre*^ mice 63% of CRF^–^ neurons tested displayed a trend to fire action potential spontaneously compared to 45% of CRF^+^ neurons. We found no significant difference in the firing rates of spontaneously active CRF^+^ and CRF^–^ neurons (Supplementary Figure 2A, *right*), consistent with what has been previously reported (Hou et al., 2016). In 74% of SST^+^ neurons tested, cell-attached recordings were often accompanied by spontaneous action potential firing (Supplementary Figure 2A, *right*) as previously reported in other areas (Ibáñez-Sandoval et al., 2011; Taniguchi et al., 2011; Lewin et al., 2016).

### Optogenetic Activation of Somatostatin Expressing and Corticotropin Releasing Factor Neurons

To further characterize the physiology of CRF^+^ neurons in the BRN and LC, optogenetic techniques were used to manipulate their electrophysiological activity in slices from *Sst*^*Cre*^ and *Crf^*Cre*^* mice bred with *chr2-yfp-flox* mice. We first determined the effect of light activation on CRF^+^ neurons in these transgenic mice. As shown in Supplementary Figure 2B (*left*), repetitive pulses of blue light consistently evoked sustained action potential firing in CRF^+^ neurons in the BRN. The maximal frequency evoked by light in the CRF^+^ neurons in the cell-attached mode was 16.72 ± 4.26 Hz (*n* = 14 mice, 30 cells). Upon whole-cell current clamp recording of these neurons, blue light activation induced depolarization that was capable of triggering repeated action potentials (Supplementary Figure 2B, *middle*). To assess the extent and shape of optogenetically activated currents, we employed the voltage-clamp recording mode (V_*hold*_ = −60 mV) as in the example in Supplementary Figure 2B (*right*). The peak amplitude of the ChR2 current was on average 111.25 ± 26.81 pA (*n* = 15 mice, 23 CRF^+^ cells). To compare the contribution of SST^+^ neurons toward the regulation of synaptic physiology in the BRN-LC circuitry with that of CRF^+^ neurons, we light activated ChR2 expressing neurons in brainstem slices of *Sst^*Cre*^;chr2-YFP* mice. In the BRN, optogenetic activation of SST^+^ neurons using repetitive light pulses as in the example in Supplementary Figure 2C, consistently evoked sustained action potential firing in extracellular loose seal recordings from SST^+^ neurons in the BRN area (*n* = 5 mice, 11/11 cells).

### Synaptic Responses in Barrington’s Nucleus and Locus Coeruleus Neuron From Activation of ChR2 Expressing Somatostatin Expressing Neurons

**FIGURE 2:**
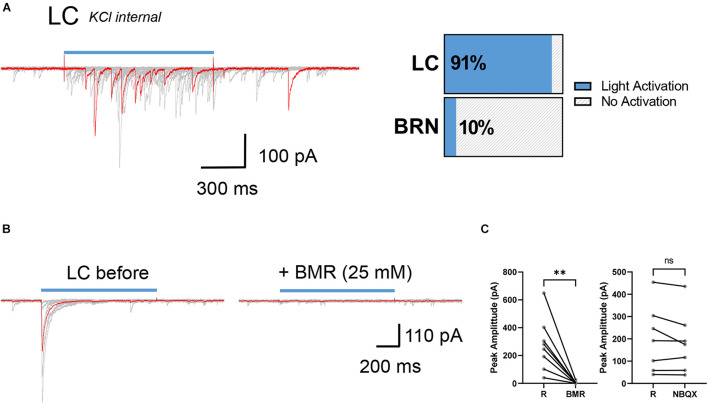
Synaptic connectivity between SST^+^ neurons and LC cells in *Sst^*Cre*^;chr2-yfp* mice. **(A)** Example superimposed (red and gray) current traces recorded from a SST^–^ neuron in the LC (KCl internal, V_*hold*_ = –60 mV) after repetitive stimulation of SST^+^ presynaptic afferents with light *(left)*. Horizontal slices chart represents percentage of postsynaptic SST^–^ neurons activated by light in LC and BRN *(right)*. LC: *n* = 10 mice, 30/33 cells light activated; BRN: 3 mice, 1/10 cells light activated. **(B)** Example superimposed (red and gray) light activated IPSCs recorded from a LC neuron in the presence and absence of BMR (25 μM). **(C)** Paired before-after plots of light-evoked IPSC in the absence (R) and the presence of BMR (*left*, *n* = 3 mice, 8 cells; R: 277 ± 67 pA; BMR: 5.5 ± 3 pA) and the absence (R) and presence of NBQX (*right*, *n* = 3 mice, 7 cells; R:199 ± 56 pA; NBQX: 182 ± 51 pA) mean ± SEM. **p ≤ 0.01; ns, not significant. Wilcoxon matched-pairs signed rank test.

We then compared light activated synaptic responses between BRN and LC neurons in brain slices of *Sst^*Cre*^;chr2-yfp* mice (*n* = 13). Since we were expecting SST^+^ neurons to be GABAergic as in other brain areas, recordings were made with high intracellular KCl at a holding potential of −60 mV to maximize the detection of postsynaptic GABA-mediated chloride currents avoiding membrane depolarization required when low internal chloride is used. Only in 1 of 10 BRN cells from 3 *Sst^*Cre*^;chr2-yfp* mice we observed light evoked IPSCs that were small and unreliable (not shown). To increase the likelihood of synaptic release we used 30 μM AP. This procedure did not increase the detection of IPSCs in BRN neurons but it increased the occurrence of spontaneous bursts of IPSCs. In striking contrast in 30 of 33 LC neurons from 10 *Sst^*Cre*^;chr2-yfp* mice light-activation robustly increased the occurrence of spontaneous and light evoked IPSCs with variable peak amplitude (Figure 2A). 4AP perfusion strongly increased the occurrence of spontaneous and light-evoked bursts of IPSCs in LC neurons in *Sst^*Cre*^;chr2-yfp* mice. However, the light-evoked IPSCs in 4AP were so large that they often produced unclamped spiking and were not measured. Exposure to the GABA_A_ receptor antagonist, BMR (25 μM) abolished both the spontaneous and light-evoked IPSCs (Figures 2B,C). In contrast, the AMPA receptor antagonist NBQX (5 μM) failed to alter evoked-synaptic responses in LC neurons (Figure 2C, *right*) implying that light-evoked IPSCs were not generated by glutamate released by intermediary neurons in polysynaptically pathways.

**FIGURE 3:**
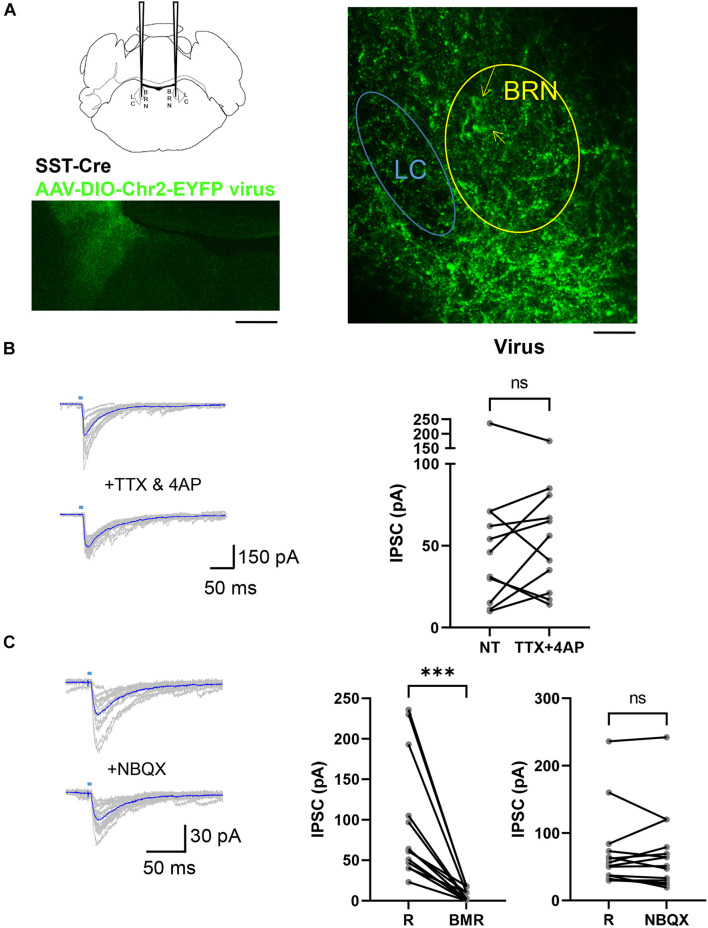
AAV DIO ChR2 Virus injections in the Pons of *Sst*^*Cre*^ mice. **(A)** *Sst*^*Cre*^ mice were injected in the pontine region, labeled BRN in the schematic (*left, top*), with a *cre*-dependent virus. Low magnification illustration of the resulting ChR2-EYFP expression (*left, bottom*) in the BRN 3 weeks after injection of AAV-EF1a-DIO-EYFP virus that expresses EYFP in cre expressing neurons (*right*). Yellow arrows point at the soma of YFP expressing SST^+^ neurons in BRN. The location of LC was verified by the presence of a cluster of larger neurons in DIC as in Figure 1 and the location of TH staining. Scale bar = 200 μm and 100 μm. **(B)** To the *left* are superimposed examples of current traces of light-evoked synaptic currents in a LC neuron voltage clamped at –60 mV recorded in an AAV-EF1a-DIO-EYFP virus injected mouse before (*top*) and during (*bottom*) the application of TTX (1 μM) and 4AP (30 μM). Traces in blue are averages of at least 10 repetitive light stimulations. To the *right* is a paired before-after plot of light-evoked IPSC in the absence (R) and the presence of TTX + 4AP (*n* = 3 mice, 11 cells; R: 58 ± 19; TTX + 4AP: 60 ± 14) mean ± SEM. ns, not significant. Wilcoxon matched-pairs signed rank test. **(C)** Example current traces from voltage clamped LC^–^ neurons in an AAV-EF1a-DIO-EYFP virus injected mouse in the absence (*top*) or presence (*bottom*) of perfusion with NBQX at –60 mV. Paired before-after plots of light-evoked IPSC in the absence (R) and the presence of BMR (*middle*, *n* = 4 mice, 14 cells; R: 92 ± 20 pA; BMR: 6 ± 2 pA) and the absence (R) and presence of NBQX (*right*, *n* = 4 mice, 14 cells; R: 72 ± 16 pA; NBQX: 70 ± 16) mean ± SEM. ***p ≤ 0.001; ns, not significant. Wilcoxon matched-pairs signed rank test.

To assess the possibility that SST terminals expressing ChR2 on LC neurons derive from distal brain regions like the Central Nucleus of the Amygdala which sends neurons that innervate this pontine region (McCall et al., 2015; Ye and Veinante, 2019) we used a viral approach. *Sst^*Cre*^* mice (4–8 weeks) were injected with AAV5-EF1a-DIO-hChR2(H134R)-EYFP viruses that enabled the expression of ChR2-EYFP in *Cre* expressing neurons in the pons. As shown in Figure 3A, SST^+^ neurons and their terminals were labeled by this procedure with EYFP in the BRN-LC area. Using pontine slices from these mice we investigated light evoked IPSCs in LC neurons. To further assess the location of neurons producing IPSCs, we combined 4AP (30 μM) with TTX (1 μM), while employing light stimulation to the pontine area encompassing the whole BRN and LC. This ChR2-assisted circuit mapping approach, CRACM (Petreanu et al., 2007, 2009), enables one to photo-stimulate ChR2-positive axons terminals even if they are severed from their parent soma. Of the 11 LC neurons recorded from 3 mice, 4AP + TTX did not affect light-evoked IPSCs (Figure 3B, *right*). In most cells studied, we did not observe failures due to the large amplitude of response that was maintained in TTX and 4AP. In the absence and presence of TTX and 4AP the latency was significantly increased from 4.81 ± 0.45 ms to 6.37 ± 0.68 ms (*p* < 0.01, paired *t*-test). The jitter was not significantly changed from 1.17 ± 0.20 ms to 1.36 ± 0.20 ms with TTX and 4AP (*p* = 0.4121, paired *t*-test). Consistent with Petreanu et al. (2009), these results show that desynchronization and temporal smearing of the light-evoked synaptic current across different synaptic terminals occurs in the presence of TTX and 4AP. These results together with the lack of change of the coefficient of variation of the amplitude (0.436 ± 0.05 ms, 0.526 ± 0.05 ms, *p* = 0.1577, paired *t*-test), suggest multiple SST inputs converging at individual LC neurons. Perfusion with NBQX in 14 LC neurons from 4 mice did not significantly affect light evoked IPSC (Figure 3C, *right*) while BMR abolished them (Figure 3C, *middle*). These results taken together suggest that optogenetic stimulation of SST^+^ neurons in BRN release GABA from SST neurons located in the pons onto LC neurons but not on those in the BRN.

### Synaptic Responses in Barrington’s Nucleus and Locus Coeruleus Neuron From Activation of ChR2 Expressing Corticotropin Releasing Factor Neurons

We then examined the synaptic responses of optogenetic activation of CRF^+^ neurons. Since BRN CRF^+^ neurons are reportedly glutamatergic (Hou et al., 2016; Keller et al., 2018), we used a potassium gluconate based intracellular solution at a holding potential of −60 mV to maximize detection of light-evoked excitatory synaptic responses. As seen in Partridge et al. (2016), CRF^+^ neurons activation in the amygdala of *Crf^*Cre*^;chr2-yfp* mice can induce reciprocal synaptic activation superimposed to the large ChR2 current in CRF^+^ neurons. However, in BRN, a light elicited increase in fast spontaneous EPSCs evoked by reciprocal excitation was observed only in 1 out of 23 CRF^+^ neurons (16 mice). Supplementary Figure 3A shows these EPSCs after subtraction from each individual sweep of light evoked currents of the average underlying ChR2 current. In addition, this was observed only in the presence of 4AP (30 μM), which enhances synaptic transmission. No light evoked IPSCs were observed when voltage clamping these neurons at −35 mV to distinguish inward synaptic currents from those that are outward (not shown).

In CRF^–^ neurons (*n* = 7 *Crf^*Cre*^;chr2-yfp* mice, 12 cells), we failed to observe either light-induced EPSCs or IPSCs while voltage clamping the neurons at −60 or −35 mV (not shown). Perfusion with 4AP (30 μM) increased the frequency of sEPSCs that was accompanied by occasional bursting (Supplementary Figure 3B left). Light activation of BRN in the presence of 4AP produced an increase in small EPSCs only in one CRF^–^ neuron (Supplementary Figure 3B, *middle*) and IPSCs in another neuron (Supplementary Figure 3B, *right*) out of 10.

Recordings were also obtained from LC neurons in the *Crf^*Cre*^;chr2-yfp* transgenic mice (*n* = 10 mice, 21 cells). To enhance synaptic activity, the cells were exposed to 4AP, which increased the frequency of both sEPSCs and sIPSCs (Supplementary Figure 3C). Light elicited action potentials and EPSCs frequency increased in only four neurons (17%), but this was exclusively in the presence of 4AP, as in the example in Supplementary Figure 3C, NBQX (5 μM) blocked all spontaneous and light-evoked currents in all LC neurons tested (data not shown). Supplementary Figure 3D reports the number of cells tested in a graphical form. The sectioning plane of the pons has been reported to affect the integrity of the dendritic tree of the LC neurons (Travagli et al., 1996), however, this was not the case as a few and unreliable light-evoked EPSCs were seen only in one of six LC cells in horizontal slices and in the presence of 4AP.

We also studied light-evoked synaptic responses in *Crf^*Cre*^* mice that were injected with a Cre-recombinase dependent virus at 5 weeks postnatal. First, we compared the expression of tdTomato in *Crf^*Cre*^;tdTomato* mice with the cre dependent viral induction of EYFP in Cre expressing BRN neurons using AAV-DIO-EYFP virus. As seen in Supplementary Figure 3E, tdTomato and YFP were broadly co-localized; although not completely, suggesting some discrepancy between the distinct approaches. Cell-attached recording of light activation with brief (5 ms) light pulses produced action potential bursting in BRN CRF^+^ neuron in slices from a *Crf^*Cre*^* mouse injected with the AAV-DIO-ChR2-EYFP virus. However, in contrast to the response seen in *Crf^*Cre*^;chr2-yfp* CRF^+^ neurons (see Supplementary Figure 2B) sustained light application of increasing intensity easily caused depolarization block of BRN CRF^+^ neurons in virus injected *Crf*^*Cre*^ mice (Supplementary Figure 3F). The whole cell ChR2 current was on average 259 ± 87 pA (*n* = 2 mice, 6 cells). This was significantly larger than that seen in CRF^+^ neurons of *Crf^*Cre*^;chr2-yfp* mice (111 ± 27 pA; *n* = 16 mice, 23 neurons; *p* ≤ 0.01, Mann–Whitney test). Light activation of synaptic responses was not observed in these mice (CRF^+^ = 0/6 cells; CRF^–^ = 0/6 cells; LC = 0/4 cells.

### Pseudorabies Virus Injection Labeling of Barrington’s Nucleus and Locus Coeruleus Neurons From the Bladder

**FIGURE 4:**
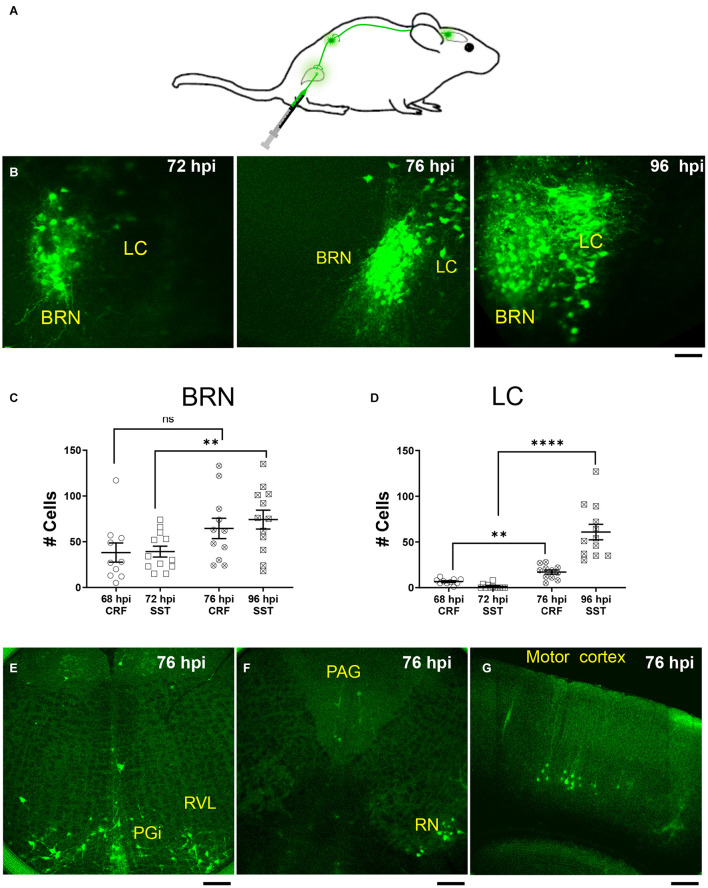
PRV expression in BRN and LC after urinary bladder injections. **(A)** Schematic of injection and retrograde labeling pathway from bladder to brainstem of PRV-152 EGFP. **(B)** Example confocal z stack projections illustrating fluorescently labeled neurons in the pontine area following injection of the retrograde *trans-*synaptic tracer, PRV-152 EGFP into the detrusor muscle of the urinary bladder after 72 h (*left*), 76 h (*middle*), and 96 h (*right*) post injections (hpi, hours post injections). Scale bar = 100 μm. **(C)** Summary of the result of PRV labeled cell count in the BRN (68 hpi CRF BRN: empty circles, *n* = 1 mouse, 4 slices, 10 BRN areas, 38 ± 11 cells; 72 hpi SST BRN: empty squares, *n* = 2 mice, 5 slices, 12 BRN areas, 39 ± 6 cells; 76 hpi CRF BRN: crossed circles, *n* = 1 mouse, 3 slices, 11 BRN areas, 65 ± 11 cells; 96 hpi SST BRN: crossed squares, *n* = 2 mice, 6 slices, 12 BRN areas, 74 ± 10 cells). **(D)** Summary of the result of PRV labeled cell count in the LC (68 hpi CRF LC: empty circles, *n* = 1 mouse, 4 slices, 10 LC areas, 6.7 ± 0.96 cells; 72 hpi SST LC: empty squares, *n* = 1 mouse, 5 slices, 12 LC areas, 1.3 ± 0.72 cells; 76 hpi CRF LC: crossed circles, *n* = 1 mouse, 4 slices, 11 LC areas, 17 ± 2.3 cells; 96 hpi SST LC: crossed squares, *n* = 2 mice, 6 slices, 12 LC areas, 61 ± 8.5 cells). **(C,D)** Areas measured from 50 μm thick confocal Z stacks (each area refers to the right or left BRN/LC area of the slice and/or the left or right LC/BRN area on the other side of slice) mean ± SEM, ns, not significant, **p ≤ 0.01, ****p ≤ 0.0001, two-tailed Mann–Whitney test. **(E–G)** Illustration of PRV-152 EFGP labeled neurons at 76 h post injection in more rostral and caudal slices to BRN. PGi, paragigantocellularis nucleus; RVL, Rostroventrolateral nucleus; RN, Red Nucleus; PAG, periaqueductal gray nucleus. Scale bar = 100 μm **(E,F)**, 200 μm **(G)**.

To determine whether CRF^+^, CRF^–^, and SST^+^ neurons in the pontine area are bladder-related, we injected the retrograde *trans-*synaptic tracer, PRV-152 EGFP into the detrusor muscle of the urinary bladder in *Crf^*Cre*^;tdTomato* and *Sst^*Cre*^;tdTomato* mice (Figure 4A). As can be seen in Figure 4B, virus labeling was present in the BRN, but not LC 72 h after PRV-152 EGFP injection into the bladder (Figure 4B, *left*). However, at 96 h post-injection PRV labeling was observed in larger size LC neurons (Figure 4B, *right*). The result of PRV labeled cell count in BRN and LC at 68, 72, 76, and 96 h post injections are summarized in Figures 4C,D, respectively. The cell count of PRV labeled BRN neurons at 96 h in an *Sst^*Cre*^;tdTomato* mouse was significantly increased compared to the time point at 72 h, this was not the case for the cell count of PRV labeled BRN neurons at 76 and 68 h from a *Crf^*Cre*^;tdTomato* mouse (Figure 4C). The number of PRV labeled LC neurons at 76 and 96 h were significantly increased compared those at 68 and 72 h, respectively (Figure 4D). LC neurons at 76 h were labeled at the same time (Figures 4E–G) as brain areas including those nuclei known to be anatomically linked to BRN or LC such as paragigantocellularis nucleus, rostroventrolateral nucleus, red nucleus, periaqueductal gray nucleus, and even in the motor cortex (Samuels and Szabadi, 2008; Van Bockstaele et al., 2009; Dimitrov et al., 2013). At 96 h, the number of cells labeled in these areas increased and additional nuclei displayed label cells (not shown).

A comparison of the distribution of PRV-labeled neurons (green) with the respect to either CRF^+^ (red; Supplementary Figures 4A,B) or SST^+^ neurons (red; Supplementary Figures 4C,D) in the BRN/LC area at different post-PRV-injection time-points revealed that CRF^+^ neurons in BRN were widely labeled by PRV as early as 68 h post injection, and that the majority of PRV labeled neurons were CRF^+^.

SST^+^ neurons were only labeled in BRN by PRV at later time-points similar to PRV labeling of LC neurons (Supplementary Figures 4C,D). Notably, dual labeled SST^+^ and PRV neurons were relatively rare in BRN and were not seen in LC.

## Discussion

Nuclei in the dorsal pons regulate autonomic functions and arousal. Using *Crf^*Cre*^* or *Sst^*Cre*^* mice we optogenetically stimulated CRF and SST neurons in BRN, the pontine micturition center, to study synaptic interactions in the region of BRN and the arousal-related norepinephrine-containing nucleus, LC. The major findings of our study, are as follows: (1) *trans-*synaptic virus labeling from the urinary bladder first infects neurons in BRN that are primarily CRF^+^, followed by LC neurons and later by SST^+^ neurons; (2) optogenetic activation of SST^+^ neurons in the BRN robustly influences synaptic physiology in the LC via GABAergic neurotransmission; (3) optogenetic activation of CRF^+^/glutamatergic neurons produces weak excitatory synaptic activation in the BRN and LC in conditions of enhanced excitability.

### Somatostatin Expressing Neurons of Barrington’s Nucleus

Although SST neurons were a relatively small and scattered population of cells in the BRN, they are positioned to influence either BRN or LC neurons. This contrasted with the lack of PV neurons in this area. To determine whether SST^+^ neurons were part of the micturition circuitry, we injected the *trans-*synaptic tracer, PRV-152 EGFP in the detrusor muscle of the bladder in *Sst^*Cre*^;tdTomato* mice. The substantially delayed labeling of SST^+^ neurons compared to CRF^+^ neurons suggests that they are relatively removed from bladder regulation. Our observation that PRV injected into the bladder of *Crf*^*Cre*^;*tdTomat*o or *Sst^*Cre*^;tdTomato* mice first labels BRN neurons, followed by LC neurons (Figure 4), and last SST^+^ neurons (Figure 4 and Supplementary Figure 4) suggest that the SST^+^ neurons are synaptically part of the BRN micturition circuitry, and suggests that the LC may project to the BRN. While this conceivable, it must be interpreted with caution as the LC has several efferent CNS targets that include amongst others the rostroventral medulla (Samuels and Szabadi, 2008), which also displayed PRV label at 76 h post injection (Figure 4). LC could be labeled from the paragigantocellularis nucleus or medullary areas, as have been previously reported in rat (Rouzade-Dominguez et al., 2003). Nevertheless, at the longer post-PRV injection period (96 h post injection) label was found to extend to the midbrain and forebrain areas such as the hypothalamus, amygdala, and cortex; thus further confirming the complexity of the stimuli that impinge on the pontine micturition center similarly to what has been reported (Yao et al., 2018).

Surprisingly, optogenetic activation of SST^+^ neurons had little effect on BRN neurons, while producing strong and reliable IPSCs in LC neurons. The failure to abolish light-evoked IPSCs by the AMPAR antagonist suggests that SST^+^ inhibition of LC neurons is direct and not mediated by a polysynaptic pathway. Notably, our data also demonstrate that SST^+^ neurons are GABAergic as evident by blockade of the light-evoked synaptic activity by BMR. Thus SST^+^ neurons in the pontine area may be pivotal in the recent finding of an active control of arousal by a locus coeruleus GABAergic circuit (Breton-Provencher and Sur, 2019). While we acknowledge the existence of SST^+^ neurons outside the pontine micturition center should also be considered, our data showing selective light activation of SST^+^ terminals in virally transduced SST^+^ neurons in the Pons are in line with the findings that a dense population of GABAergic neurons located in the LC region, visualized by locally injecting Flox-ChR2 virus in *Gad^*Cre*^* mice, forms functional synaptic contacts with and inhibits LC-NA neurons (Breton-Provencher and Sur, 2019). IHC staining for GAD in *Sst^*Cre*^;tdTomato* mice supported these findings.

### Corticotropin Releasing Factor Neurons of Barrington’s Nucleus

Barrington’s nucleus neurons project to the lumbosacral spinal cord to innervate preganglionic parasympathetic neurons that regulate bladder contraction during the micturition reflex. Dual retrograde labeling studies suggested that a subpopulation (approximately 10%) of these neurons also project to the LC, providing a potential means by which this nucleus could coordinate bladder contraction with arousal in response to bladder filling (Valentino et al., 1996). The *Crf^*Cre*^;tdTomato* mice used in the present study showed a similar localization of CRF^+^ neurons in the dorsal pons as described in previous studies (Valentino et al., 1995; Hou et al., 2016; Keller et al., 2018). Notably, only about 50% of the CRF^+^ neurons were bladder-related and this is similar to the percentage reported in studies using retrograde tract tracing and CRF^–^ immunohistochemistry (Valentino et al., 1995).

In forebrain nuclei that have abundant CRF^+^ neurons such as the paraventricular hypothalamic nucleus, bed nucleus of the stria terminalis and central nucleus of the amygdala, optogenetic activation of CRF neurons influences the activity of both CRF^+^ and CRF^–^ neurons in the region (Hou et al., 2016), indicative of regulation of intranuclear activity by CRF. In contrast, in the present study, activation of CRF^+^ neurons within BRN did not result in consistent excitatory (or inhibitory) synaptic responses of either CRF^+^ or CRF^–^ BRN neurons. The occasional synaptic responses observed were only seen in the presence of 4AP, which increases synaptic release and induces bursting activity. This suggests that their generation in BRN neurons was via engagement of internal or external polysynaptic networks. Moreover, the light-evoked EPSCs and IPSCs could be only observed in a few neurons even in the presence of 4AP, which suggest that the collaterals of BRN CRF^+^ neurons are not critical to these polysynaptic pathways. The paucity of BRN CRF^+^-induced synaptic activation could be indicative of a lack of sufficient expression of ChR2 in the CRF^+^ neurons with cross-breeding from birth. However, this was unlikely because even in slices from adult mice in which the cre-dependent AAV-DIO-ChR2-EYFP virus-transduced CRF^+^ neurons in the BRN, optogenetic activation similarly failed to elicit light-induced synaptic responses. In these virally transduced neurons, the light-induced current was not only doubled but strongly controlled their firing frequency and also easily caused a depolarization blockade in them. Importantly, the two approaches label a similar number and distribution of CRF^+^ neurons in the BRN.

A subpopulation of spatially clustered glutamatergic CRF^–^ BRN neurons that express estrogen receptor 1 have been reported, which innervate interneurons of the spinal cord responsible for inhibition of the urethral sphincter (Keller et al., 2018). In the BRN, the CRF^–^ neurons are closely interspersed with the CRF^+^ neurons (Valentino et al., 1995; Hou et al., 2016). This proximity of the two neuronal types suggests that they may be synaptically connected. However, the present findings showing a lack of effect of CRF^+^ activation on CRF^–^ cells argue against this organization. It is likely that the coordination of the two BRN neuronal populations to influence bladder-sphincter function occurs elsewhere in the CNS or at the level of the spinal cord.

Barrington’s nucleus neurons are strategically positioned to form collateral connections with neurons in the adjacent LC nucleus that initiates arousal (Rizvi et al., 1994) and retrograde tracing studies suggest that a subpopulation of CRF^–^ BRN neurons collateralize to both the LC and spinal cord (Valentino et al., 1996). This structural organization would allow the BRN to coordinate the elimination of urine with an arousal response. Recordings of LC and cortical activity during cystometry in unanesthetized rats demonstrate that as the bladder fills, LC activity anticipates peak micturition pressure (Manohar et al., 2017). Approximately 30 s prior to peak bladder pressure, LC neurons become activated and cortical activation occurs simultaneously. Coherence between the LC and cortex in theta frequency increases at this time and may serve as a code to initiate and promote voiding behaviors. The temporal offset between the shift in LC-cortical network activity may allow time to disengage from unrelated ongoing behaviors prior to initiating micturition and allow the animal to urinate in safe and socially appropriate conditions (Manohar et al., 2017). These findings provided a rationale for investigating the influence of BRN input to LC neurons in the pontine slice preparation. Surprisingly, optogenetic activation of CRF^+^ neurons in the BRN of *Crf^*Cre*^;chr2-yfp* mice did not produce consistent excitatory postsynaptic currents in LC. This was true both in coronal and horizontal slices. A similar lack of consistent effects was seen in adult *Crf^*Cre*^* mice injected with AAV-DIO-ChR2-EYFP virus even though the light-induced current was doubled and in the presence of 4AP. Interestingly, BRN recordings during cystometry in unanesthetized rats also suggest that the consistent with the BRN driving LC and cortical activity. Indeed, in a case in which BRN and LC neurons were recorded in the same animal, LC activity usually preceded BRN activity (Manohar et al., 2017). Together, these results argue against an organization whereby BRN neurons coordinate arousal and micturition and suggest that this may be regulated by another region that projects to both nuclei, such as the periaqueductal gray region. Alternatively, it is possible that the subpopulation of BRN neurons that is hypothesized to project to the LC based on retrograde tracing studies is not detected in recordings or sufficiently activated in optogenetic studies.

In summary, the present study shows that CRF^+^ neurons in the BRN have a relatively minor impact on the synaptic activity of the LC. In contrast, GABAergic SST^+^ neurons in this pontine micturition center are a substantial source of influence that support the hypothesis of a contribution to the regulation of behavior via the LC.

## Data Availability Statement

The raw data supporting the conclusions of this article will be made available by the authors, without undue reservation.

## Ethics Statement

The animal study was reviewed and approved by the Institutional Animal Care and Use Committee, Georgetown University.

## Author Contributions

SG, NS, JV, SZ, RV, and SV contributed to the conception and design of the study. SG, DC, HK, JV, SZ, NS, RV, and SV performed the experiments, analyzed the data, prepared the figures, and drafted, edited, and revised the manuscript. All authors contributed to the article and approved the submitted version.

## Conflict of Interest

The authors declare that the research was conducted in the absence of any commercial or financial relationships that could be construed as a potential conflict of interest.

## Publisher’s Note

All claims expressed in this article are solely those of the authors and do not necessarily represent those of their affiliated organizations, or those of the publisher, the editors and the reviewers. Any product that may be evaluated in this article, or claim that may be made by its manufacturer, is not guaranteed or endorsed by the publisher.
