# Supplementary material for: Somatostatin Neurons in the Mouse Pontine Nucleus Activate GABAA Receptor Mediated Synaptic Currents in Locus Coeruleus Neurons
Source: Front Synaptic Neurosci. 2021 Oct 4;13:754786. doi: 10.3389/fnsyn.2021.754786 (PMC8524133; doi:10.3389/fnsyn.2021.754786)
Supplement: Supplementary file 1 [file Data_Sheet_1.PDF]

Supplementary Material

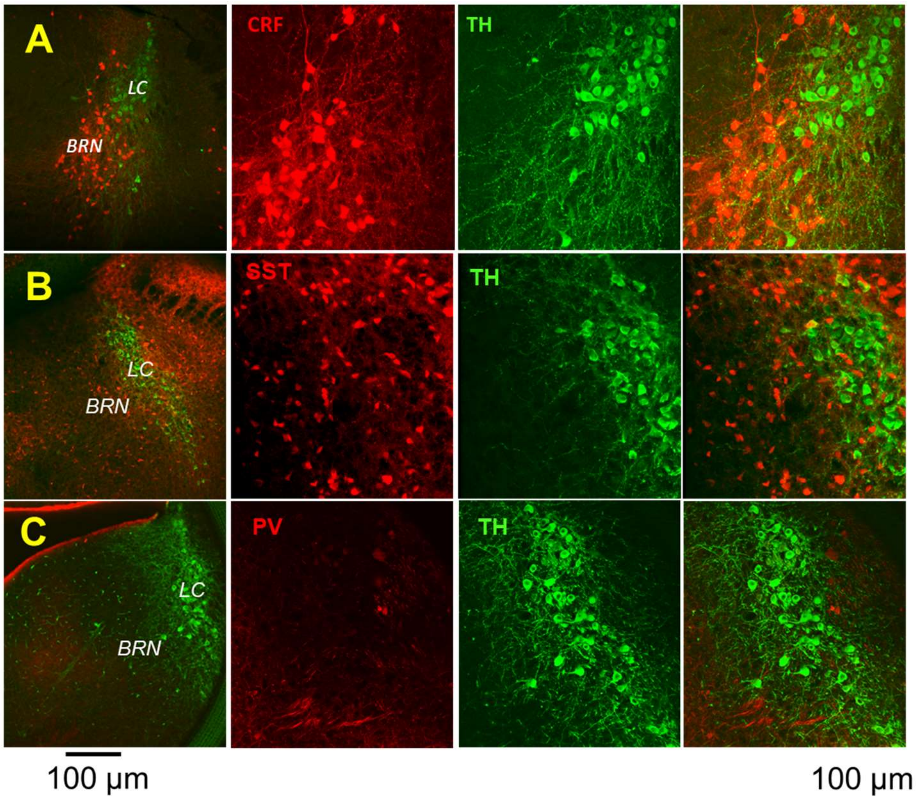

Supplementary Figure 1.1. Neurons in Barrington's Nucleus/LC area of *in Crf*^{Cre}; *Sst*^{Cre}; and P*V*^{Cre}*; tdTomato* mice. Immunodetection of TH neurons of the LC in coronal brains slices of *Sst*^{Cre}*;tdTomato* **(A)**, *Crf*^{Cre};*tdTomato* **(B)** or *Pv*^{Cre};*tdTomato* mice. Low magnification fluorescence images illustrate the extent of fluorescence in slices containing the BRN and LC (*left*). Anatomical distribution is compared at a higher magnification between CRF **(A)**, SST **(B)** or PV **(C)** mediated tdTomato expression (red), anti-TH antibody staining (green), and merged images (yellow).

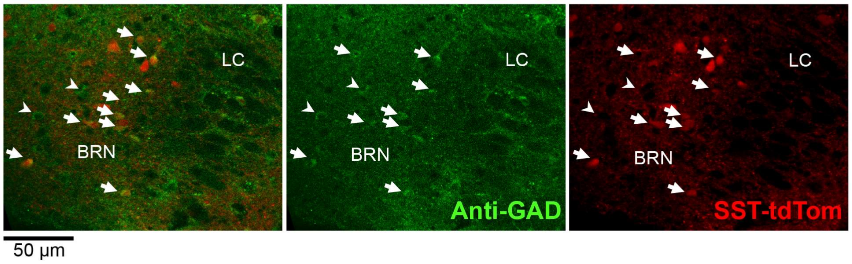

Supplementary Figure 1.2. GAD immunostaining in the BRN-LC area of a *Sst*^{Cre}*;tdTomato* mouse. GAD^{+} neurons in the BRN, labeled anti-GAD (green) colocalized with the expression of tdTomato, labeled SST-tdTom (red, white arrows). Occasional GAD^{+} neurons that did not express tdTomato were also observed (red, arrow heads).

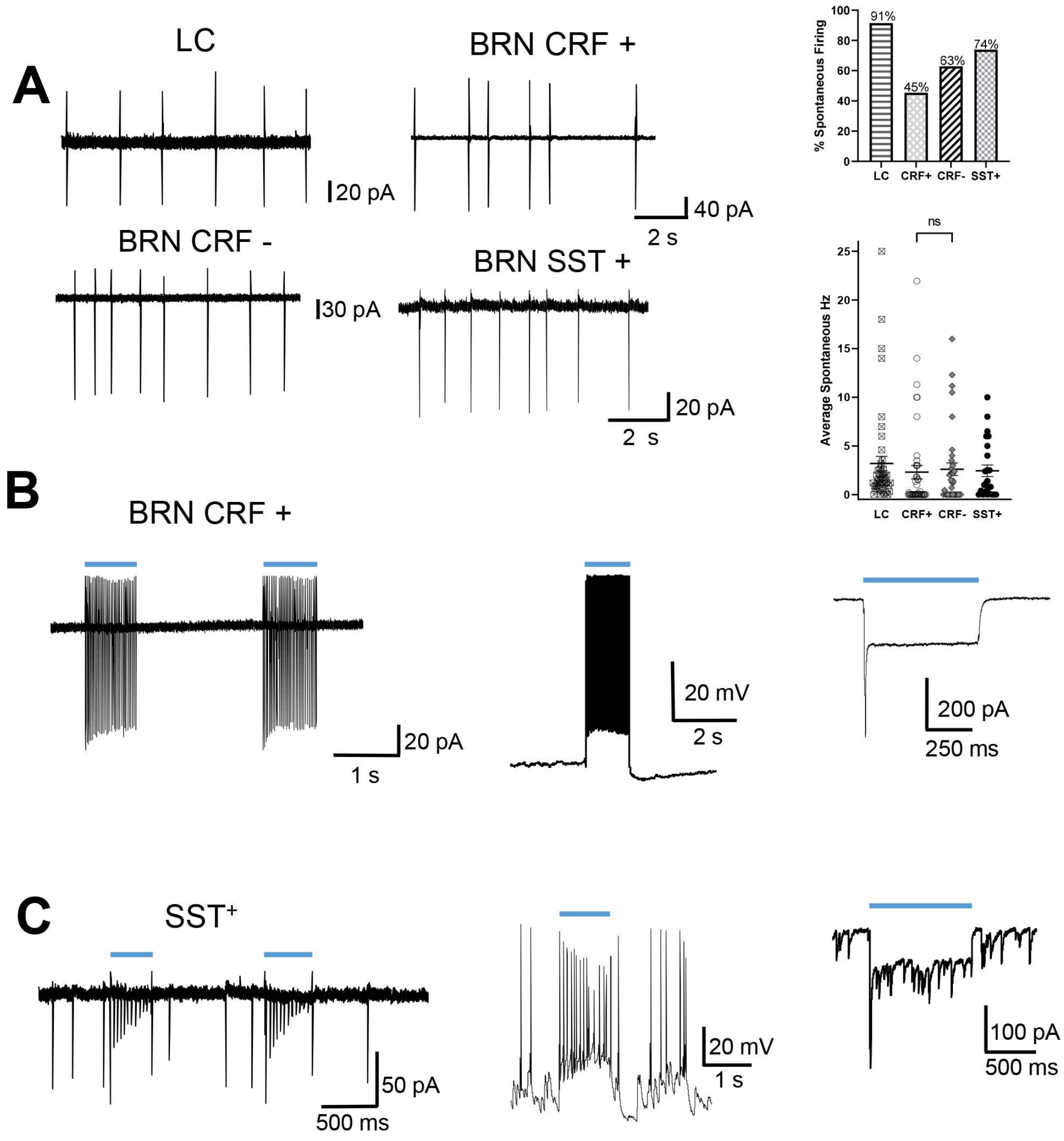

Supplementary Figure 2. Electrophysiology of CRF$^+$ and SST$^+$ neurons in BRN and LC. **(A)** Example cell-attached recordings from visually identified LC and CRF$^+$, CRF$^-$, and SST$^+$ neurons in the BRN illustrating spontaneous action potential firing (*left*). To the right are shown a summary of % of cells with spontaneous firing (*top*) and the average frequency of firing (*bottom*). LC: n= 17 mice, 43/47 cells spontaneous, 3.20$\pm$ 0.73 Hz; BRN CRH$^+$: n= 19 mice, 20/44 cells spontaneous, 2.31$\pm$ 0.69 Hz; BRN CRH$^-$: n= 11 mice, 22/37 cells spontaneous, 2.61 $\pm$ 0.65 Hz; BRN SST$^+$:n = 10 mice, 17/24 cells spontaneous, 2.45$\pm$ 0.60 Hz. Black bars, mean. ns: not significant (p=0.2276), two-tailed Mann-Whitney test. **(B)** Example trace of repetitive action potentials recorded in cell-attached from a CRF$^+$ neuron in the BRN evoked consistently with repetitive pulses of blue light indicated by blue trace (*left panel*). Action potentials triggered by depolarization induced by blue light in a CRF$^+$ neuron in whole-cell current clamp recording of example BRN neuron (*middle panel*). Optogenetically activated current from an example BRN CRF$^+$ neuron in a voltage clamp recording (V_{hold} = -60 mV, *right panel*). **(C)** Representative trace of sustained action potential firing in SST$^+$ neurons evoked by blue light in extracellular loose seal recording (*left*), whole cell current clamp, and (*middle*). Voltage clamp recording (V_{hold} = -60 mV, *right*).

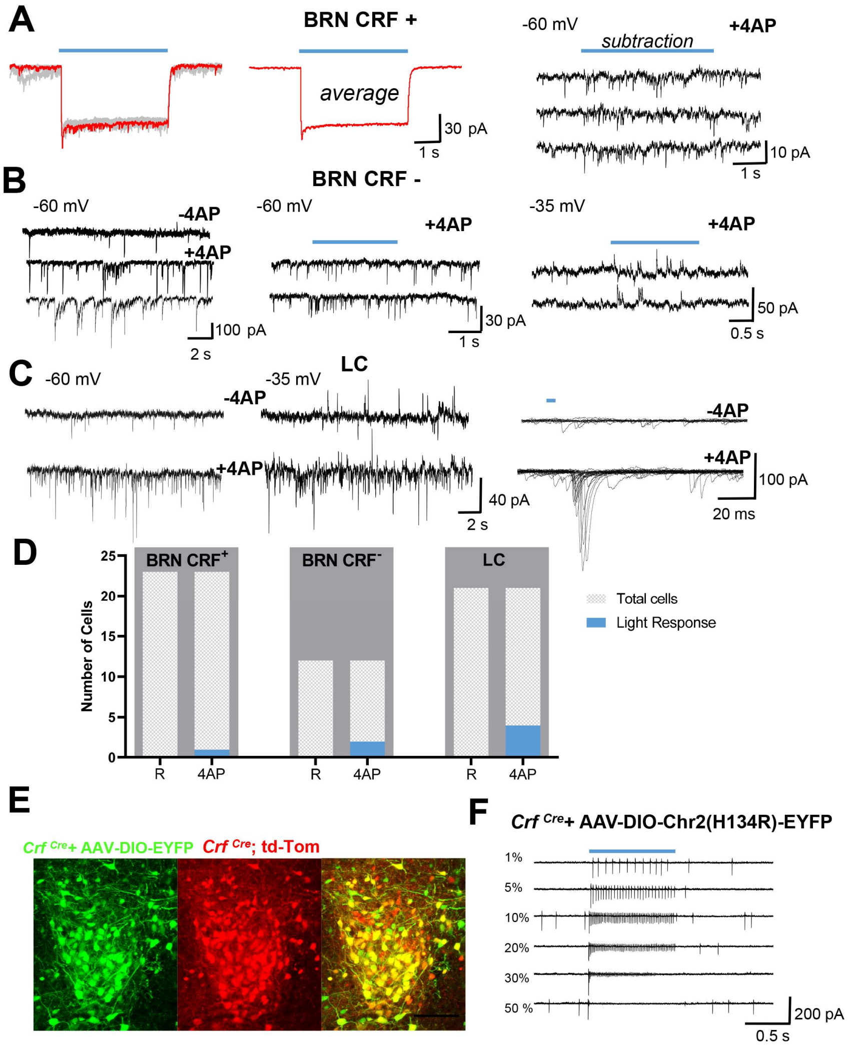

Supplementary Figure 3. Synaptic connectivity between CRF^{+}, CRF^{-} and LC cells in response to light. **(A)** (*left*) Light evoked ChR2 currents with overlaying synaptic currents (sEPSCs) in a BRN CRF^{+} ChR2 neurons in the presence of 4AP (30 µM); (*middle*) average of 10 responses to blue light activation. (*right*) sEPSCs frequency is increased by light as seen after subtraction of the average ChR2 current from each individual light response, see also (10). **(B)** (*left*) Example current traces from voltage clamped BRN CRF^{-} neurons in the absence (*top trace*) or presence (*middle & bottom traces*) of perfusion with 4AP; light activation of sEPSCs at -60 mV (*middle*) or sIPSCs at -35 mV (*right*) in distinct BRN CRF^{-} neurons. **(C)** Voltage clamp recordings of spontaneous synaptic activity in the absence (*top traces*) or presence *(bottom traces)* of 4AP in LC neurons with pulse of blue light held at -60 mV (*left*) or -35 mV (*middle*); (*left*) light-evoked EPSCs in the absence (*top traces*) or presence *(bottom traces)* of 4AP. **(D)** Distribution of total cells recorded in the pons with light-activated responses (EPSCs or IPSCs) in the absence (R) and the presence of 4AP. CRF^{+}: n = 16 mice, 0/23 cells R, 1/23 cells 4AP; CRF^{-}: n = 7 mice, 0/12 cells R, 2/10 cells 4AP; LC: n = 10 mice, 0/21 cells R, 4/21 cells 4AP. (**E**) *Crf*^{Cre} mediated tdTomato expression (red, *middle*) in the BRN from *Crf*^{Cre};*tdTomato* mouse 3 weeks after injection of AAV-EF1a-DIO-EYFP virus that express EYFP in *Cre* expressing neurons (green, *left*). Merged images illustrated substantial overlapping (yellow, *right*) Scale bar = 100 µm **(F)** light application of increasing intensity (% maximal LED intensity), caused depolarization block of action potential firing in a BRN CRF^{+} neuron in a slice from 2 months old *Crf*^{Cre} mouse injected with AAV-EF1a-DIO-hChR2(H134R)-EYFP virus.

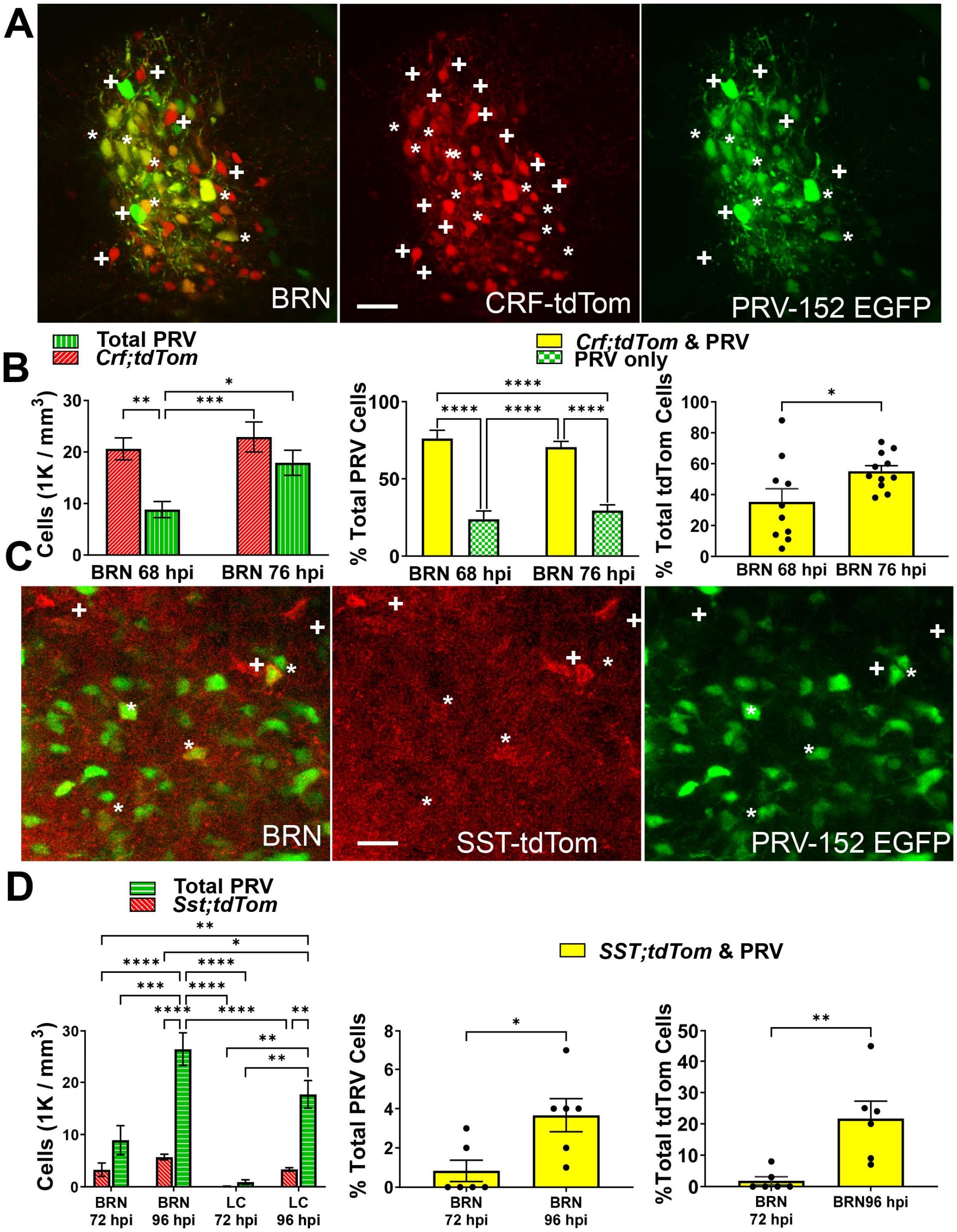

Supplementary Figure 4. CRF^{+} and SST^{+} neurons retrogradely labeled by PRV. Example confocal z stack projections illustrating fluorescently co-labeled PRV-152 EGFP and C*rf*^{Cre}*;tdTomato* **(A)** or *Sst*^{Cre}*;tdTomato* **(C)** expressing neurons in BRN. Red tdTomato fluorescence (*middle*) and green EGFP fluorescence (*right*) are also shown superimposed (*left*). (*) identify co-labeled neurons, (+) identify neurons only expressing CRF, SST, or PRV. (**B**) (*left*) Summary of PRV and tdTomato cell counted in slices from PRV injected *Crf*^{Cre}*;tdTomato* mice expressed as cells density (1000/mm^3) normalized to the volume examined (average 50 planes confocal Z stacks at 1 µm step size x the estimated area of BRN in each confocal plane) at 68 hours post-injection (hpi) (n=1 mouse, 4 slices, 10 BRN areas) and 76 hpi (n=1 mouse, 3 slices, 11 BRN areas). BRN 68 hpi: *Crf*;tdTom: 21± 2.1cells (1K/ mm^3); PRV: 8.8±1.6 cells (1K/ mm^3). BRN 76 hpi: *Crf*;tdTom: 23±2.9 cells (1K/ mm^3);

PRV: 18±2.4 cells (1K/ mm^3). (*middle*) Percentage of CRF^+ tdTomato labelled cells also labeled with PRV (solid yellow) over all PRV labeled cells counted in BRN (tdTomato & PRV, 68 hpi: 76±5.3 %; 76 hpi: 71± 3.7%) and percentage of CRF^- PRV only cells over all PRV labeled cells counted in BRN (PRV only, 68 hpi: 24±5.3 %; 76 hpi: 29±3.7 %). (*left &middle*) Two-way ANOVA followed by Tukey's multiple comparisons test. (*right*) Percentage of CRF^+ tdTomato cells also labeled with PRV (solid yellow) over all tdTomato labeled CRF^+ cells in the BRN (68 hpi: 35±8.5 %; 76 hpi: 55±3.6 %) at the two time points considered. Two-Tailed Mann Whitney test. **(D***)* (*left*) Summary of PRV and tdTomato positive cells counted in slices from PRV injected *Sst*^{Cre}*;tdTomato* mice in the BRN and in the LC at 72 and 96 hours post-injection expressed as in B. BRN 72 hpi (n= 1 mouse, 2 slices, 6 BRN areas): *Sst;tdTom*: 3.3±1.3 cells (1K/ mm^3); PRV: 8.9±2.8 cells (1K/ mm^3). LC 72 hpi (n= 1 mouse, 2 slices, 4 LC areas): *Sst;tdTom*: 0.08±0.05 cells (1K/ mm^3); PRV: 0.90±0.44 cells (1K/ mm^3). BRN 96 hpi (1-2 mice, 2-6 slices, 6-12 BRN areas): *Sst;tdTom*: 5.67± cells (1K/ mm^3); PRV: 26±3.2cells (1K/ mm^3). LC 96 hpi (1-2 mice, 2-6 slices, 6-12 LC areas): *Sst;tdTom*: 3.3±0.35 cells (1K/ mm^3); PRV: 18±2.7 cells (1K/ mm^3). Two-way ANOVA followed by Tukey's multiple comparisons test. (*middle*) Percentage of SST^+ tdTomato labeled cells also labeled with PRV (solid yellow) over all PRV labeled cells counted in BRN (72 hpi: 0.83±0.54 %; 96 hpi: 3.7±0.84 %) and (*right*) SST^+ tdTomato labeled cells also labeled with PRV over all tdTomato labeled SST^+ labeled cells counted in BRN(72 hpi: 1.8±1.3 %; 96 hpi: 22±5.6 %) at the two time points considered (n= 1 mouse, 2 slices, 6 BRN areas). Two-tailed Mann Whitney test. Scale bar = 50 µm. mean±SEM,,*:p ≤0.05, **: p≤0.01,***: p≤0.001 ****: p≤0.0001.
